# Reconcentrating the Ionic Liquid EMIM-HSO_4_ Using Direct Contact Membrane Distillation

**DOI:** 10.3390/molecules30020211

**Published:** 2025-01-07

**Authors:** Mark J. Wong, Viral Sagar, Joan G. Lynam

**Affiliations:** Department of Chemical Engineering, Louisiana Tech University, 600 Dan Reneau Drive, P.O. Box 10348, Ruston, LA 71272, USA; mwong4095@gmail.com (M.J.W.);

**Keywords:** 1-ethyl-3 methylimidazolium hydrogen sulfate, DCMD, polytetrafluoroethylene, polyvinylidene, conductivity, UV-vis

## Abstract

Adequate water supplies are crucial for missions to the Moon, since water is essential for astronauts’ health. Ionic liquids (ILs) have been investigated for processing metal oxides, the main components of lunar regolith, to separate oxygen and metals. The IL must be diluted in the process. Recycling this diluted IL post-processing is important to reduce the materials required in resupply missions. In addition, water will be needed in lunar greenhouses for growing food and aiding in sustaining a habitable environment. Direct contact membrane distillation (DCMD) is a new technology for water purification that was examined in this study for its feasibility to concentrate IL. Hydrophobic membranes composed of polytetrafluoroethylene (PTFE) and polyvinylidene (PVDF) were found to hold promise in separating solutes from water to concentrate a diluted IL solution and to recover water. A bench-scale DCMD system was employed to test this method at temperatures of 50 °C, 65 °C, and 80 °C. Hence, the benefits and limitations of DCMD with PTFE and PVDF membranes were explored for the aqueous IL 1-ethyl-3 methylimidazolium hydrogen sulfate for DCMD performed at different temperatures.

## 1. Introduction

As humans venture to explore deep space, we must surmount many challenges. Some of the greatest challenges are primary life support systems needed to ensure the long-term survival of astronauts working during extended missions. Functional life support systems must be capable at least of providing oxygen, water, and CO_2_ removal [1,2]. Meeting these challenges is a significant hurdle in extraterrestrial exploration. Recycling processes are vital, but no process will have 100% efficiency, so there always will be the necessity to produce new water and oxygen. Using electrolysis, oxygen can be produced from water, similarly to methods used on military naval vessels, but liquid water is rare in our region of space, with most lunar water being concentrated as ice at the Moon’s poles [3]. Various technologies have been proposed to deal with this concern in non-polar areas. One potential solution suggested has been separating metal oxides to access the embedded oxygen and then reacting it with hydrogen to create water [4]. This method is thought to be viable due to the discovery that the Moon contains large quantities of various metal oxides, such as iron oxide and titanium oxide [5].

A new approach to meeting oxygen requirements on the Moon is employing ionic liquids (ILs) to process metal oxides common in lunar regolith to create water by using the oxygen from metal oxides and hydrogen, which can be recycled. One particular IL, 1-ethyl-3 methylimidazolium hydrogen sulfate, has permitted the operating temperature for this process to be reduced to less than 200 °C [6]. Water, however, will be introduced into and dilute the IL. The IL solution must be concentrated to remove the water before it is re-used. The separated water could then be converted with electrolysis to generate oxygen and permit the hydrogen gas to be recycled back into the system. Direct contact membrane distillation (DCMD) was this work’s selected method for exploring this process. DCMD has potential for removing water from the diluted IL stream, allowing the IL to be concentrated during the process and lessening the need for system purging.

DCMD is a new method for separating water from aqueous solutions. DCMD has been suggested for processes such as desalination plants and dairy processing. The basis for the system is a temperature difference between hot aqueous solutions being passed on the bottom of a thin and porous hydrophobic membrane, with a cold water stream being passed on the top [7]. During the DCMD process, the temperature gradient creates a vapor pressure difference. The polymer membrane enables water vapor to pass through from the high-temperature stream to condense in the low-temperature water stream. The membrane’s hydrophobic nature would be expected to allow only water vapor to pass through the membrane, owing to the IL solution being repulsed by the membrane or the solute molecules being too big to pass through the membrane. DCMD’s main advantage is that it does not require the IL solution to be completely vaporized. The complexity of the process is thus reduced significantly, decreasing the construction volume of the module and/or the energy requirements. This is in contrast with the more common method of separation, in which all or most of the liquid is vaporized and then condensed to produce a solute-rich base and a water-rich condensate. Typical distillation permits some contaminants to be entrained in the condensate [8]. Traditional distillation methods work by either providing energy, in the form of heat, to the solution to vaporize it or by reducing the pressure with a vacuum system to cause vaporization at a lower temperature [7]. Nevertheless, these operations have serious drawbacks, since heat-based vaporization is energy-intensive and also requires extra equipment. Vacuum-based methods require a complex system of interconnected pumps that may vary in operation and individual process control systems, which require either a batch-based method or a complex process to allow for continuous processing, increasing possible failure points.

DCMD provides the ability to separate water from aqueous streams in a manner that is energy-efficient and has reduced spatial requirements [9]. DCMD separations do not require full vaporization, so complex, large distillation tower systems are not needed for water reclamation. DCMD also provides the benefits of vacuum distillation without its complexity, because recycled or waste heat sources could be used. For these reasons, the amount of energy that needs to be fed into the system could be reduced, increasing the energy efficiency of the process [7].

A few membrane-based distillation methods have shown adequate results since the 1960s [10]; however, their implementation in the past was considered ineffective due to issues with polymer technology. The underlying basis of the DCMD method relies on hydrophobic polymer-based membranes, with their effectiveness drastically shifting the effectiveness of the process. Advances in membrane and polymer technology have expanded the useful applications of membrane-based systems, but technological issues still remain that must be addressed. Typical concerns include the membranes’ costs and quality. Membrane quality difficulties can result in the degradation of the membrane, with rates dependent on the type of aqueous solution being processed and the quantity that is being pumped across the membrane. More research is required to understand mass and energy transfer across membranes with reference to varying molecule sizes in the solution [10].

The present work studied the ability of a DCMD system in separating water from a diluted IL solution, since concentrated IL will be needed for use in acquiring the oxygen in metal oxides from lunar regolith [6]. Little information on using DCMD on diluted IL is available in the literature, so this research is novel [11]. Two of the most commonly used membranes for DCMD are polytetrafluoroethylene (PTFE) and polyvinylidene (PVDF), so these membranes were chosen for the experimental work [12].

## 2. Results and Discussion

### 2.1. Conductivity Calibration

**Figure 1:**
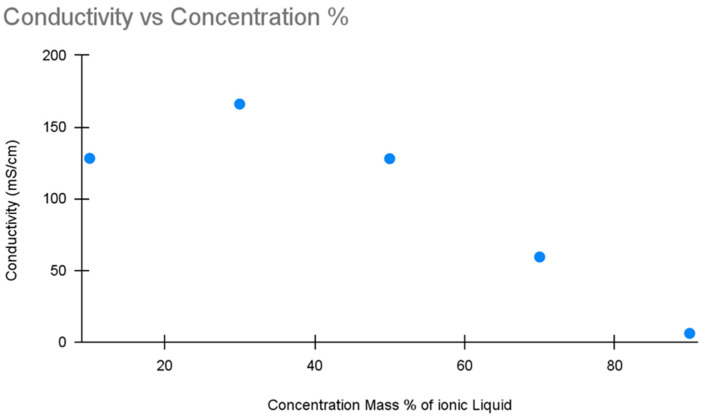
Calibration curve to establish the relationship between the conductivity and mass percentage of ionic liquid (IL).

The data on the conductivity and concentration of IL displayed a nonlinear relationship. The polynomial relationship between these is shown in Figure 1.

The conductivity of 1-ethyl-3 methylimidazolium hydrogen sulfate versus concentration was found to be of a parabolic form, as seen in Figure 1. Other researchers have found similar a parabolic form for another IL *n*-(2-hydroxyethyl)piperazinium propionate [13]. Starting at the y-axis with water and then adding IL initially increases the conductivity, possibly due to the ions moving and breaking up hydrogen bonding in the water. As more ions are added, more hydration shells of water molecules form around the ions and the ions may eventually form their own lattice structure [14], causing less mobility and thus lower conductivity. ILs similar to 1-ethyl-3 methylimidazolium hydrogen sulfate have frequently been described as viscous, likely due to intermolecular ionic bonds reducing mobility and thus conductivity [15].

Table 1 shows that at the higher DCMD temperatures, the PTFE membrane permitted an increase in contamination in the water product stream. While these contaminations were low, this finding still represents a consideration for use in long-term water purification methods. This finding also suggests that there is some transfer rate of ions across the membrane that is outpaced by the transfer of water vapor. It is probable that there is an optimum operating temperature differential needed between the two streams to ensure that the rate of water passing through the membrane outstrips the rate of the solute. However, with DCMD using the PVDF membrane, little contamination of the water stream with ions occurred.

UV-Vis spectroscopy was used along with conductivity to provide more reliable data. The conductivity data supplemented the UV-Vis spectroscopy results.

### 2.2. Absorption Calibration

**Figure 2:**
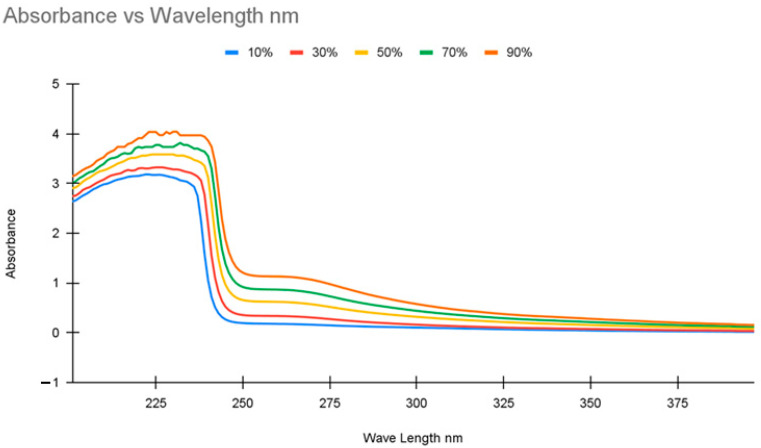
Calibration curve to establish the relationship between mass percentage of IL and light absorption.

The typical calibration curve, as shown in Figure 2, for the UV-Vis spectroscopy was as expected for solutions made primarily of two non-water components. Two peaks were seen in the ultraviolet range. The first peak collection was found at ~238 nm and the second presented at ~265 nm. The first peak (238 nm) was neglected since all samples resulted in absorbance peaks greater than 2, conditions that reduce the quality of the data, with the light sensor beginning to become overloaded. Instead, the second peak (265 nm) was used as the primary measuring tool.

**Figure 3:**
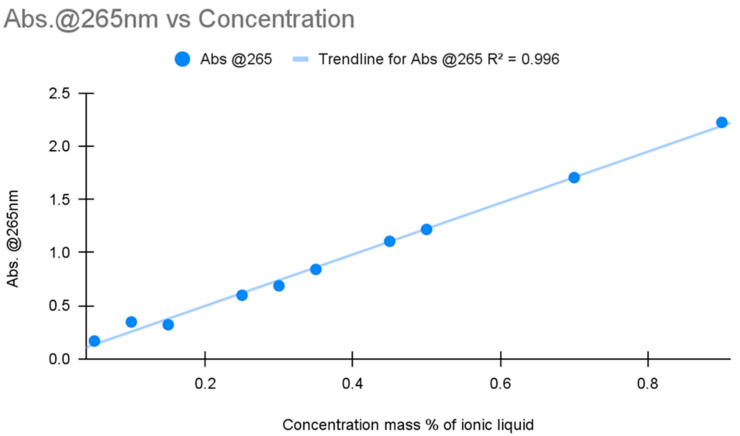
A calibration curve for the absorbance of light at 265 nm for different mass percentages of IL.

A graph was then produced with the absorption of light at 265 nm for both calibration curves and then combined for linear quantification, as shown in Figure 3. UV-Vis spectra were obtained for all samples taken in the experiment’s 12 trials.

The concentration of IL and respective absorbance values are seen to be linearly related. These findings allowed all needed calculations to be performed.

Table 1 and Table 2 reveal the overall change in the IL solution based on the membrane material and temperature of the run. Table 2 and Table 3 show that at 80 °C, the average water flux across the PTFE membrane appears to be almost double that of the PVDF membrane.

**Figure 4:**
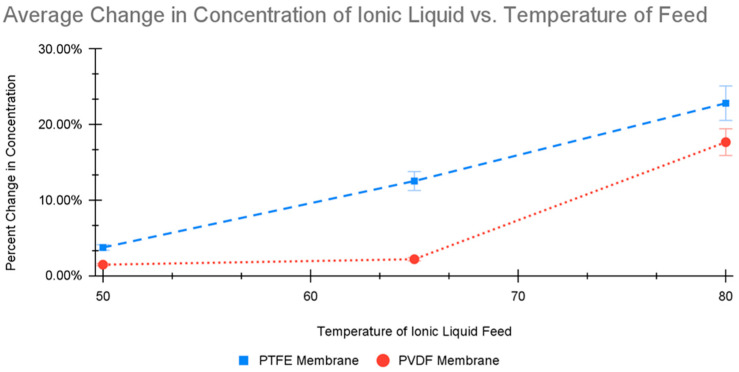
The effect of DCMD temperature on the change in concentration of the IL solution.

The change in results is most apparent when the two membranes are compared. The PTFE membrane was much more effective at transferring water from the feed stream. For 80 °C operation, the average water flux across the PTFE membrane was greater than that of the PVDF membrane. Figure 4 shows that both the PVDF and PTFE membranes transfer water through the membrane in a somewhat polynomial fashion. This was expected because the vapor pressure of water has a polynomic form. Nonetheless, the PTFE membrane appears to be much more linear. Also, the concentration of the IL changed more when the PTFE membrane was used compared to the PVDF membrane.

PTFE membranes appear to transfer water at a higher rate at the equivalent temperatures compared to their PVDF counterparts. However, this comes with the drawback of greater permeate contamination. Testing the conductivity of the final water stream confirmed that slight leakage must have occurred (Table 1). A possible explanation for this is the pore size difference between the two membranes. The PTFE membrane had a pore size of 0.45 microns, while the PVDF membrane had a 0.1-micron pore size. The pore size difference could explain the change in distillate contamination rates.

## 3. Materials and Methods

The IL tested was 1-ethyl-3 methylimidazolium hydrogen sulfate with a stock concentration of 95% (Sigma-Aldrich, St. Louis, MO, USA, S/N:49615761, CAS # 412009-61-1). Water content was assumed to be 5%. For the scope of this research project, it was assumed that the processing of metal oxides from lunar regolith had already been conducted and that it had successfully resulted in an aqueous IL solution. The solution’s initial desired concentration was chosen to be 20%. This percentage was chosen because it was the concentration used in previous studies [6].

The solutions were processed using DCMD. The setup included a heated IL solution peristaltically pumped underneath the bottom of the polymer membrane and cooled water peristaltically pumped above the top layer of the polymer membrane. The temperatures of the two solutions (cooled and heated) were kept constant using water baths. The temperature of the cooled water was kept constant using a water bath at 5 °C. The heated water bath’s temperature was varied to 50 °C, 65 °C, and 80 °C. The reading error for temperature was ±1 °C, while the accuracy of the temperature reading was 0.1 °C. Two runs were performed at each temperature for 2 h with feed rates of 200 mL/min on each side of the membrane. After two tests at each of the three operating temperatures, the membrane used was changed from a PTFE to a PVDF polymer-based membrane (Sterlitech, Kent, WA, USA: 0.45 Micron PTFE Flat Sheet Membrane, with Polypropylene Netting Backer Laminated, Sepa, Lot#: J000014897 11-1; and Novamem 0.1 Micron Flat Sheet Membrane, PVDF100, MF, Sepa, Lot#: 624096PVDF100). The operation was then replicated to test how different membranes would interact with the IL.

During DCMD, 50 mL samples were removed at 30 min intervals from the hot IL solution. Samples were also taken initially before beginning the process and after it had finished. The final water obtained from the cold side was retained to test for the transfer of ions across the membrane. The mass of water from the cold side before and after each experiment was weighed and recorded. This was performed to find the total flux of the water during the experiment. It was observed that for the trials with the PTFE membrane, after 90 min of operation the change in the solution was negligible at the lowest temperature and therefore the DCMD operation was only performed for 90 min. The experimental duration was increased to 2 h when the data recorded indicated that an increase in time was required to reach significant changes with increased feed temperature. An analysis of samples was performed with two methods, conductivity and UV-Visible spectroscopy analysis.

Conductivity tests were performed using 50 mL samples and read from an Ohaus brand conductivity meter (Parsippany, NJ, USA: Model ST20C-B). All samples were created by diluting the stock solution with water prepared with reverse osmosis. An initial calibration curve was created using a 5-point calibration curve ranging from 10% to 90% concentrations at 20% intervals. The DCMD samples were then tested using the same equipment and methodology.

UV-Visible spectroscopy (UV-Vis) functions by sending a stable beam of light through a sample to examine the change in the absorbance of the light beam after it passes through the sample. The light beam used varies within a selected range of wavelengths. The changes in absorbance at different wavelengths depend on the compound being tested. As the concentration of compounds in a sample increases, absorbance increases. Thus, concentrations for samples with compounds that absorb light can be measured quantifiably.

UV-Vis spectroscopy was performed on a Shimadzu spectrometer model no. UV-2401PC (Shimadzu Co., Kyoto, Japan) with a blank water sample as a baseline, and then 3 mL samples were used in quartz cuvettes. Two initial calibration curves were performed. The initial calibration was performed with the same solutions used to calibrate the conductivity meter, five solutions ranging from 10 to 90% concentration spaced out at 20% intervals. The second calibration was performed using five solutions ranging from 5 to 45% concentration spaced at 10% intervals. In the Supplementary Materials, Figures S1–S4 give more detailed UV-Vis data.

## 4. Conclusions

Overall, the tests performed indicate the potential for DCMD technology to be employed in operating a water recovery system for ILs after use in lunar regolith processing. Both membranes tested offered advantages and drawbacks for use in water recovery depending on the water permeate’s end purpose. For purposes where the water quality can be flexible, the PTFE membrane operating at a high temperature would be preferable. These DCDM operating conditions offer a larger change in the concentration of the IL, permitting a faster recovery period before it can be reused in the initial DCMD process. However, this option would have the drawback of contaminating the permeate at a significantly faster rate than for the PVDF counterpart. The disadvantage of water contamination would have to be considered. In situations when high water quality must be maintained, such as for drinking water, the PVDF membrane would be preferred. This membrane gave a decreased water recovery rate but also provided a lower rate of contamination of the permeate. Both membranes did offer viability in the ability to recover water from ILs that have been diluted, resulting in a product stream of increased IL concentration.

Studying the life span viability of a variety of membrane materials will be essential in future work. Sulfuric acid has been reported to increase the pore size of PVDF membranes when exposed to it over a period of weeks [16]. Hydrogen sulfate or bisulfate is considered a weaker form of sulfuric acid. When incorporated in an IL, hydrogen sulfate’s activity may be curtailed due to bonds with 1-ethyl-3 methylimidazolium [17]. This study showed the viability of PTFE and PVDF membranes for performing the task of recovering water from IL streams. If put into practice for lunar missions, the resupply of IL and membranes could be reduced. Even so, a significant quantity of membranes would be required. This can only be defined by understanding the effective life spans of the membranes. This study had each membrane operate for approximately 12 h per membrane. In an industrial-level application, these membranes will need to be tested with the IL on the order of weeks of continual operating time

## Figures and Tables

**Table 1:** Change in water conductivity (mS/cm) based on feed temperature and membrane type.

Feed Temp (°C)	ΔConductivity PTFE mS/cm	ΔConductivity PVDF mS/cm
50.0	165.0	14.8
65.0	1162.0	85.8
80.0	1560.9	259.4

**Table 2:** Total average difference in solution properties with the PTFE membrane at various temperatures.

Temperature °C	Total Water Exchange Grams	Average Flux g/m^2^·h	Change in Concentration
50	4.61	0.16	3.71%
65	14.98	534.82	12.52%
80	44.66	1595.00	22.83%

**Table 3:** Total average change in solution properties with polyvinylidene (PVDF) membrane at various temperatures.

Temperature °C	Total Water Exchange Grams	Average Flux g/m^2^·h	Change in Concentration
50	15.58	556.25	1.44%
65	3.83	136.79	2.16%
80	22.47	802.32	17.67%

## Data Availability

The original contributions presented in the study are included in the article/Supplementary Materials; further inquiries can be directed to the corresponding author.

## Supplementary Materials

The following supporting information can be downloaded at https://www.mdpi.com/article/10.3390/molecules30020211/s1: Figure S1: Absorption spectra of ionic liquid solution at various operation times for a PVDF with a feed temperature of 50 °C; Figure S2: Absorption of light at 265 nm of ionic liquid solutions at various operation times for a PVDF membrane with a feed temperature of 50 °C; Figure S3: Absorption spectra of ionic liquid solutions at various operation times for a PVDF membrane (LEFT) and PTFE membrane (RIGHT) with a feed temperature of 80 °C; Figure S4: Absorption of light at 265 nm of ionic liquid solution at various operation times for a PVDF membrane (LEFT) and PTFE membrane (RIGHT) with a feed temperature of 80 °C.
