# Supplementary material for: Reconcentrating the Ionic Liquid EMIM-HSO4 Using Direct Contact Membrane Distillation
Source: Molecules. 2025 Jan 7;30(2):211. doi: 10.3390/molecules30020211 (PMC11768014; doi:10.3390/molecules30020211)
Supplement: Supplementary file 1 [file molecules-30-00211-s001.zip › molecules-3341372-supplementary.pdf]

Supplemental Material:

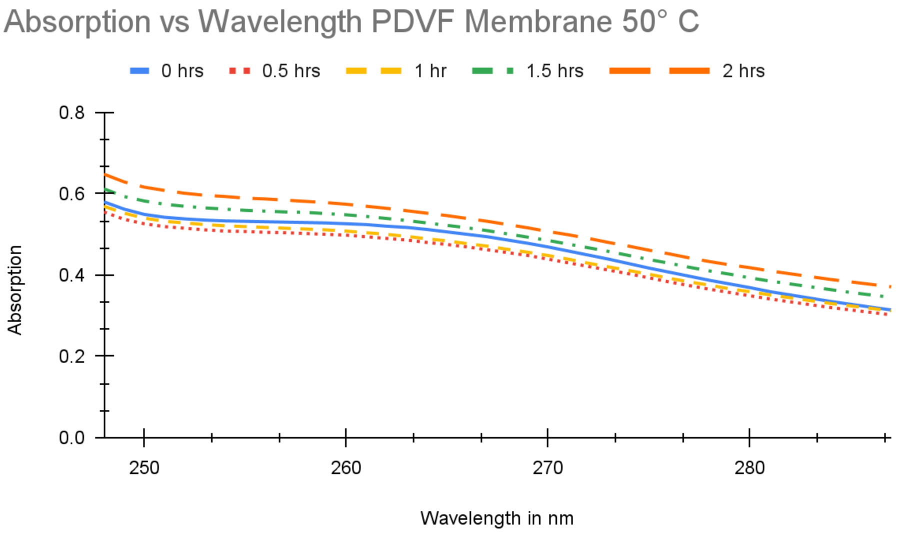

Figure S1: Absorption spectra of ionic liquid solution at various operation times for a PVDF with feed temperature of 50 ℃.

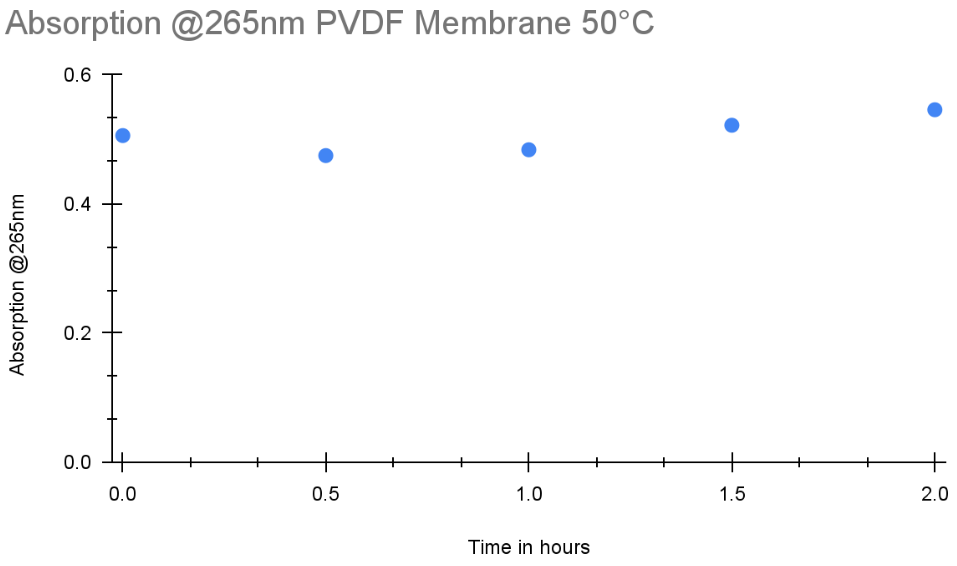

Figure S2: Absorption of light at 265 nm of ionic liquid solutions at various operation times for a PVDF membrane with feed temperature of 50 ℃.

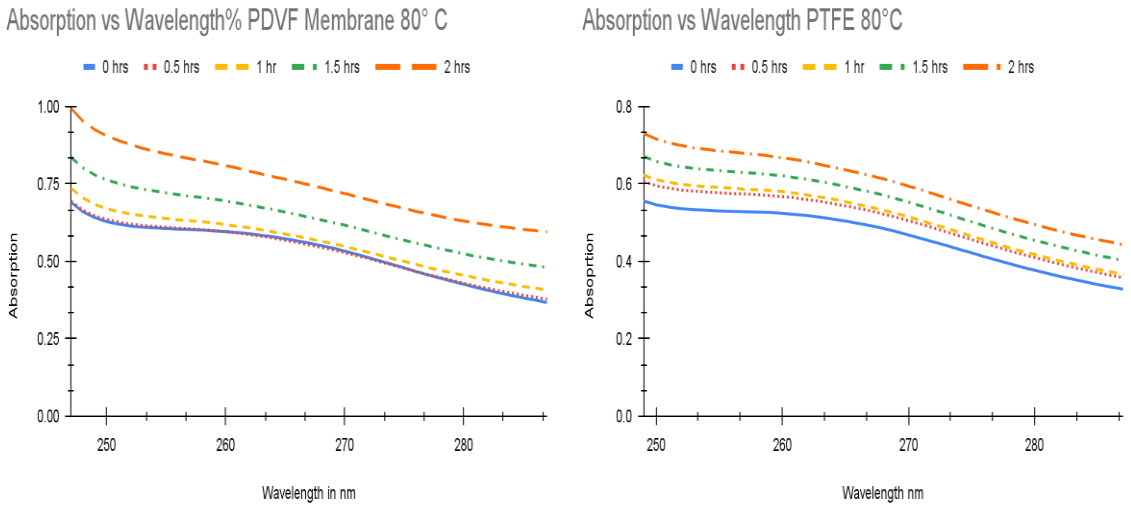

Figure S3: Absorption spectra of ionic liquid solutions at various operation times for a PVDF membrane (LEFT) and PTFE membrane (RIGHT) with feed temperature of 80°C.

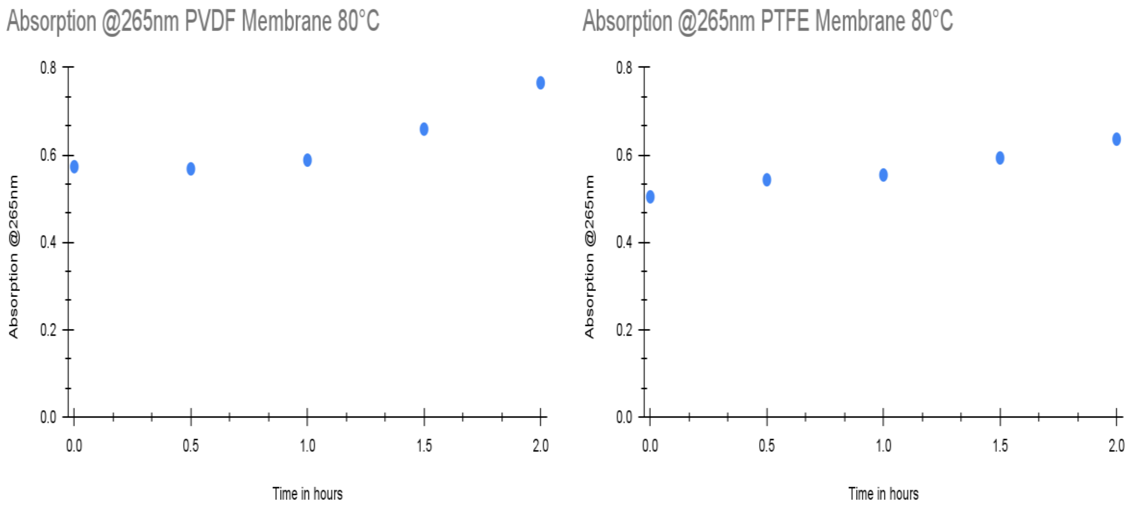

Figure S4: Absorption of light at 265 nm of ionic liquid solution at various operation times for a PVDF membrane (LEFT) and PTFE membrane (RIGHT) with feed temperature of 80°C.
